# Response to Kan et al., “A case of neonatal lupus erythematosus presenting with extensive erosions at birth, healing with extensive scarring”

**DOI:** 10.1016/j.jdcr.2024.06.042

**Published:** 2024-08-06

**Authors:** Xing-Yu Li, Jie Liu, Dong-Lai Ma

**Affiliations:** Department of Dermatology, Peking Union Medical College Hospital, Chinese Academy of Medical Sciences and Peking Union Medical College, State Key Laboratory of Complex Severe and Rare Diseases, National Clinical Research Center for Skin and Immune Diseases, Beijing, China

**Keywords:** congenital erosive and vesicular dermatosis, herpes simplex virus, neonatal lupus erythematosus

*To the Editor:* We read the article “A case of neonatal lupus erythematosus presenting with extensive erosions at birth, healing with extensive scarring” by Kan et al^1^ with great interest. However, there are several concerns related to the diagnosis that need to be discussed.

Neonatal lupus erythematosus (NLE) is an uncommon disease presented in newborns with cutaneous lesions, congenital heart block, or abnormal findings in the hematologic, hepatobiliary, and musculoskeletal systems.^2^ It is caused by the transplacental passage of maternal autoantibodies to anti-Ro(SS-A), anti-La(SS-B), and antiribonuclear protein antigens. In the case description, this patient showed extensive areas of purpuric erosions upon delivery. However, NLE skin lesions usually present between the ages of 4 and 6 weeks, rather than at birth, possibly related to photosensitivity. Second, the typical skin manifestations of NLE are erythematous annular plaques with scales, although blisters or crust can be found in severe conditions. Moreover, in NLE, the face is most often affected, followed by the scalp, trunk, and extremities, but in this case, the face was spared. Third, skin lesions of NLE can spontaneously resolve generally without scars, although telangiectasia, dyspigmentation, and slightly atrophic lesions can be observed in 27% to 34% of cases. However, this patient had extensive scarring. Fourth, although the patient had severe skin lesions, they completely healed within 3 months. However, the cutaneous lesions of NLE basically self-resolve by age 6 to 8 months as maternal IgG antibodies are gradually cleared. Fifth, almost all patients with NLE are positive for anti-SS–A and/or anti-SS–B antibodies, and antiribonuclear protein antigens less frequently. However, the patient in this case was positive for antinuclear antibody and anti-double–stranded DNA antibody, but negative for anti-Ro(SS-A) and anti-La(SS-B) antibodies. Finally, the histopathologic examination of NLE usually shows interface dermatitis with reticular dermal perivascular and periadnexal lymphocytic infiltrate, and direct immunofluorescence test result reveals IgG deposited at the dermoepidermal junction.^2^ Nevertheless, the skin biopsy of this case revealed subepidermal blister and mild basal vacuolar degeneration with red cell extravasation in the papillary and mid dermis, which are not consistent with the typical pathological changes of NLE. Direct immunofluorescence test result of the patient was negative for IgG, IgA, immunoglobulin M, C3, and fibrin.

Based on the above analysis, the diagnosis of NLE seems unlikely. We think that the diagnosis of this patient is most likely congenital erosive and vesicular dermatosis (CEVD) with reticulated supple scarring. CEVD is a rare disorder, presenting with vesicles, erythema, erosions, ulcerations, crusts, and fissures at birth and it often affects >75% of the skin surface. The skin lesions generally heal rapidly within 10 days to 3 months after birth, leaving distinctive reticulated scars with supple texture.^3^ Patients with CEVD are usually premature or small for gestational age. The skin lesions most commonly affect the trunk and limbs; however, the face, palms, and soles are generally spared.^4^ The histopathologic features of CEVD differ and depend on the stage of the disease. In the early stage, subepidermal vesiculation and eroded epidermis with various inflammatory cells infiltrated are common, especially neutrophils. In the late stage, the histopathology mainly shows scar formation, possibly with a decrease in hair follicles and absent eccrine glands.^3^ The pathogenesis of CEVD remains unknown. Intrauterine infections, amniotic adhesions, and developmental defects may participate in the pathogenesis. It is observed that about 20% of patients with CEVD have concurrent herpes simplex virus infection,^3^ but the link between herpes simplex virus and CEVD is not fully elucidated.^5^ The diagnosis of CEVD is often based on the clinical evolution and typical features of reticulated supple scarring, and the exclusion of other cutaneous diseases characterized by erosions, blisters, or vesicles at birth.

The patient was a premature infant infected with herpes simplex virus type 1. The lesions were extensively distributed over the back, buttocks, anterior aspect of the chest, arms, neck, and scalp, sparing the face. The skin lesions were present from birth but no new lesions appeared subsequently, and spontaneously self-healed within 3 months, leaving hypopigmented reticulated scars. The histopathologic results of the patient are not consistent with the typical pathological changes of NLE. Based on these factors, we believe that the diagnosis of CEVD is more appropriate for this patient.

## Conflicts of interest

None disclosed.
